# Supplementary figures and images for: An improvement in IMRT QA results and beam matching in linacs using statistical process control
Source: J Appl Clin Med Phys. 2014 Sep 8;15(5):190–5. doi: 10.1120/jacmp.v15i5.4927 (PMC5711098; doi:10.1120/jacmp.v15i5.4927)

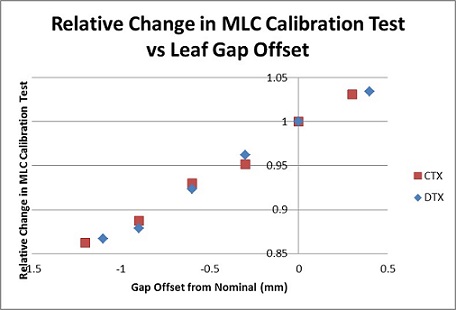

Supplement: Supplementary file 1 — Supplementary Material [file ACM2-15-190-s001.jpg]

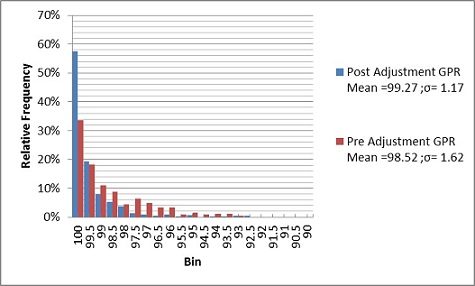

Supplement: Supplementary file 2 — Supplementary Material [file ACM2-15-190-s002.jpg]

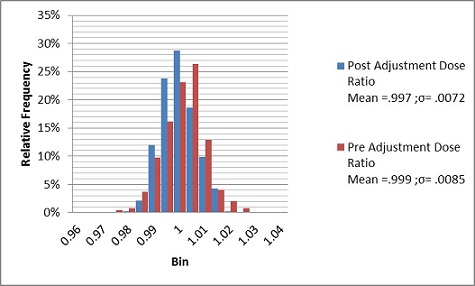

Supplement: Supplementary file 3 — Supplementary Material [file ACM2-15-190-s003.jpg]
