# Supplementary material for: An improvement in IMRT QA results and beam matching in linacs using statistical process control
Source: J Appl Clin Med Phys. 2014 Sep 8;15(5):190–5. doi: 10.1120/jacmp.v15i5.4927 (PMC5711098; doi:10.1120/jacmp.v15i5.4927)
Supplement: Supplementary file 4 — Supplementary Material [file ACM2-15-190-s004.doc]

**An Improvement in IMRT QA Results and Beam Matching in LINACs Using Statistical Process Control**

**Abstract:** The purpose of this study is to apply the principles of Statistical Process Control (SPC) in the context of patient specific Intensity Modulated Radiation Therapy (IMRT) QA to set clinic-specific action limits and evaluate the impact of changes to the Multi Leaf Collimator (MLC) calibrations on IMRT QA results. Ten months of IMRT QA data with 247 patient QAs collected on three beam matched LINACS were retrospectively analyzed with a focus on the Gamma Pass Rate (GPR) and the average ratio between the measured and planned doses. Initial control charts and action limits were calculated. Based on this data, changes were made to the leaf gap parameter for the MLCs to improve the consistency between LINACs. This leaf gap parameter is tested monthly using a MLC sweep test. A follow up data set with 424 unique QAs were used to evaluate the impact of the leaf gap parameter change. The initial data average GPR was 98.6 % with an SPC action limit of 93.7%. The average ratio of doses was 1.003 with an upper action limit of 1.017 and a lower action limit of 0.989. The sweep test results for the LINACs were -1.8%, 0%, and +1.2% from nominal. After the adjustment of the leaf gap parameter all sweep test results were within 0.4% of nominal. Subsequently, the average GPR was 99.4 % with an SPC action limit of 97.3%. The average ratio of doses was 0.997 with an upper action limit of 1.011 and a lower action limit of 0.981. Applying the principles of SPC to IMRT QA allowed small differences between closely matched LINACs to be identified and reduced. Ongoing analysis will monitor the process and be used to refine the clinical action limits for IMRT QA.

Keywords: Statistical Process Control; Beam Matching; IMRT QA

PACS Number: 87.55.Qr

**I. Introduction**

Statistical Process Control (SPC) has been used in industry since the early 1920’s to reduce waste and increase the early detection and prevention capabilities of quality control (QC) and quality assurance (QA) systems^(1)^. Recently medical physicists have begun to apply the principles of SPC to all facets of the radiation oncology clinical practice ^(2)^. Specifically, SPC can be used to identify out of control processes and improve a clinic’s intensity modulated radiation therapy (IMRT) QA program, LINAC QC program, and overall patient safety^(3-8)^. Using these methods, the medical physicist can create clinic specific QA and QC action limits that are based on real world data as opposed to more generic national guidance ^(3-7)^.

In our clinical practice we use three beam matched LINACs to treat IMRT patients. Patients are allowed to switch machines with little restriction. An analysis of IMRT performance outside the setting of daily, monthly, or annual QA had not been performed. This fact became a major motivator to study the three LINACs in detail. The ability of SPC to identify out of control processes made it an excellent tool to verify if the three LINACs were performing in a similar manner when delivering IMRT treatments. In this study we examined the initial application of SPC to our IMRT QA program, the effect of changes made to the QA program, and the long term stability of the changes.

**II. Methods**

We retrospectively analyzed ten months of IMRT QA data with 247 patient specific QAs collected on three beam matched Varian Linear Accelerators (LINACs) (21Ex and iX models) with Millennium 120 Multi Leaf Collimators (MLC). No restrictions were put on beam energy, therefore both of our clinically used photon energies, 6MV and 18MV, are represented in the pre and post adjustment datasets. We focussed on the Gamma Pass Rate (GPR) and the average ratio between the measured and planned doses. All QAs were measured as true composites with a Sun Nuclear Mapcheck 2 using the MapPhan add on, i.e. all fields were delivered at the planned gantry angles for fixed gantry plans or with the prescribed arcs, with the MapCheck in either the coronal or sagittal plane. In order to deal with the angular dependence of the MapCheck diodes, for static gantry IMRT we offset any beams entering parallel to the diode plane by at least 5^o^ in the QA plan and measurement. We make no adjustments for rotating gantry IMRT; our tests with rotating open fields indicate that the perturbation is small. The GPR was computed by comparing the measured to calculated dose planes with 3 percent, 3mm criteria. Additionally the dose threshold was set at 10% and the Van Dyk difference along with the measurement uncertainty capability of the Sun Nuclear Patient software was used. The calculated dose plane was created by mapping the patient plan onto a CT of the MapCheck 2 with Hounsfield units redefined so as to appropriately model the response. This was verified by altering the Hounsfield units until the equivalent path of the MapCheck 2 and MapPhan system as reported by Eclipse equaled 5cm. Doses were calculated with the Varian Eclipse AAA algorithm. The ratio between the measured and planned doses was calculated by averaging the results from the MapCheck software’s histogram of all data points within the 10% threshold. The standard deviation of that distribution has not been used as a quality measure because that variation is captured via the gamma distribution. As part of each IMRT QA, the MapCheck response is adjusted to match the prediction for a standard open field, thus reducing variation caused by daily output fluctuation or detector response.

Initial control charts and action limits were calculated based on methods published by Breen et al. ^(3)^. The first 25 instances of a controlled system are used to calculate the Upper Control Limit (UCL) and the Lower Control Limit (LCL).


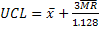
 (1)


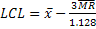
 (2)


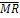
 is defined as the average of the moving range and
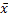
 is the mean of the data. This process was first applied retrospectively to the 247 data points obtained from IMRT QAs on all three accelerators to see if the process appeared to be in control and to determine the degree of variability.

Note that we treat the accelerators as matched and move patients between them freely, so we also do the IMRT QA test on any of the available machines. The justification for doing so depends on careful quality assurance of the MLC calibration, which we test both daily and monthly. The monthly test is performed along with the monthly output checks. A Farmer-type chamber is placed at 10 cm depth in water and the reading for a 10x10 cm^2^ field given 100 MU is obtained. Then another reading is taken with a dynamic MLC motion that sweeps a 0.5 cm gap over the 10 cm span. The ratio of the two readings depends sensitively on the gap width as shown in Figure 1, and this can be adjusted in the MLC control software by changing the leaf gap parameter.

In order to investigate possible reasons for the observed variation in the initial data set, the data was then analyzed for each accelerator separately to test whether there were systematic differences attributable to the machine, and if so, if there were concomitant variations in the monthly tests of MLC calibration that could be reduced and thereby reduce the variability in the IMRT QA results.

A follow up data set with 424 patient specific QAs was used to evaluate the effect of the MLC calibration tolerance change and the long term stability of the IMRT QA program. The followup data set has been analyzed in an identical manner as stated above.

**III. Results**

The results for the initial 247 IMRT QAs, taken prior to adjusting the tolerance of the MLC calibration test, are shown for the GPR in Figure 2 and for the average dose ratio in Figure 3. The pre adjustment average GPR was 98.6 % with an LCL of 93.7% (note that the GPR is single sided with a maximum value of 100%, so only the LCL is relevant.) The pre-adjustment average ratio of doses was 1.003 with a UCL of 1.017 and a LCL of 0.989. Table 1 shows the results in terms of means and standard deviations for the combined data set and for the three accelerators separately, along with their variation in sweep test ratio. The difference between the accelerators was statistically significant, with p values ranging from 9.3x10^-6^ to 3.9x10^-9^, and paralleled the difference in sweep test ratio. The p value for the dose was calculated using a two tailed unpaired students T test, while the p value for GPR was calculated using a one tailed unpaired students T test. Based on these findings, the tolerance of the monthly sweep test ratio was changed from 0.0730-0.0785 to 0.0739-0.0772 while the nominal target stayed constant at 0.0758. The initial sweep test tolerances were based on historical clinical experience. The new tolerances were determined by calculating the upper and lower control limits by the SPC method described above using the monthly sweep test values after the adjustment occurred.

The IMRT QA results following the adjustment are shown for the GPR in Figure 2 and for the average dose ratio in Figure 3. Post-adjustment, the average GPR was 99.4 % with an LCL of 97.3%, and the average dose ratio was 0.997 with an UCL of 1.011 and a LCL of 0.981. The differences in these results in relation to the pre adjustment values are statistically significant with a GPR p value of 3.16x10^-11^ and an average dose ratio p value of 0.0058. Table 2 shows the results for the combined and separate data sets following the adjustment. The difference between the three accelerators remains statistically significant, although the overall variability has been reduced. This strengthened our judgement that the choice of machine for QA or treatment is not clinically significant. The MLC calibration continues to be tested on a monthly basis with the sweep test and went from a pre adjustment maximum deviation of 3% to a post adjustment maximum of 1.7% from the nominal value of 0.0758. The maximum deviation for the sweep test result was calculated using a simple percent difference compared to the nominal value stated above.

**IV. Discussion**

The application of SPC to our IMRT QA program has shown many benefits both directly and indirectly. The 0.8% rise in the GPR along with the 3.6% increase of the LCL implies a distinct increase in the consistency of the IMRT QA results. The lack of improvement in the dose ratio values implies that this parameter is insensitive to the changes made to the MLC calibration. This is supported by the data in table 1 and table 2. A shift in the sweep test ratio did not result in a change to the average dose ratio. Further investigation into what accelerator parameters affect the dose ratio needs to occur as it appears to be a more complex issue. The decrease in the maximum range of the MLC calibration across the three LINACs was largely responsible for the 50% decrease in the standard deviation of the data. The MLC calibration is arguably the most important factor for consistent IMRT and VMAT performance among the three beam matched LINACs^(8-10)^. As described by Rangel et al.^(9)^ and Moiseenko et al. ^(10)^ , relatively small MLC errors can cause dosimetrically meaningful changes. These findings underscore the need to monitor and control the MLC calibration. There are still QAs that violate the control limits on a semi-regular basis. Approximately 5% of QAs fall outside of the control limits. This value has remained constant pre and post MLC calibration adjustment. Each has been flagged for individual investigation, but so far no other systematic reason for the outliers has been found.

During the period of this investigation, the proportion of IMRT treatments delivered by rotational techniques has increased from 60.7% prior to the adjustment of the sweep test tolerance to 84.9% after. It is possible that some of the reduction in variation can be attributed to this change in planning and delivery; however that has not been evaluated.

This study has not addressed the important question of what the tolerance for the gamma pass rate and dose ratio should be to declare an IMRT plan safe for delivery. However it has allowed us to reduce the variability and better identify those plans deserving further scrutiny. It is important to note that our clinical practice of performing a true composite measurement has not been altered. Therefore the gains described and the decrease in variability shows the importance of applying SPC to IMRT QA, even if the measurement system has inherent angular dependancies.

**V. Conclusion**

Applying the principles of SPC to IMRT QA allowed small differences between closely matched LINACs to be identified and reduced. Analysis has shown that the MLC calibration test results have a direct effect on the overall quality of the IMRT QA program. Declaring LINACs to be beam matched requires ongoing testing of the beam shaping devices along with the dosimetric and mechanical properties. By testing the MLC calibration and maintaining strict pass criteria, it is possible to reduce the variability in IMRT QA results and improve the quality and safety of the QA program.

**References**

1. **Pawlicki T, Mundt AJ. Quality in radiation oncology [Electronic Version]. Med Phys. 2007;34(5):1529-34.**
2. **Pawlicki T, Whitaker M, Boyer AL. Statisitcal process control for radiotherapy quality assurance [Electronic Version]. Med Phys. 2007;32(9):2777-86.**
3. **Breen SL, Moseley DJ, Zhang B, Sharpe MB. Statistical process control for IMRT dosimetric verification [Electronic Version]. Med Phys. 2010;35(10):4417-25.**
4. **Palaniswaamy G, Brame RS, Yaddanapudi S, Rangaraj D, Mutic S. A statistical approach to IMRT patient-specific QA [Electronic Version]. Med Phys. 2012;39(12):7560-70.**
5. **Mancuso GM, Fontenot JD, Gibbons JP, Parker BC. Comparison of action levels for patient specific quality assurance on intensity modulated radiation therapy and volumetric modulated arc therapy treatments [Electronic Version]. Med Phys. 2012;39(7):4378-85.**
6. **Gordon JD, Krafft SP, Jang S, Smith-Raymond L, Stevie MY, Hamilton RJ. Confidence limit variation for a single IMRT system following the TG119 protocol [Electronic Version]. Med Phys. 2011;38(3):1641-48.**
7. **Gerard K, Grandhaye JP, Marchesi V, Kafrouni H, Husson F, Aletti P. A comprehensive analysis of the IMRT dose delivery process using statistical process control (SPC) [Electronic Version]. Med Phys. 2009;36(4):1275-85**
8. **Beyer GP. Commissioning measurements for photon beam data on three TrueBeam linear accelerators, and comparison with Trilogy and Clinac 2100 linear accelerators. J Appl Clin Med Phys. 2012;14(1):273-88. Retrieved December 9, 2013 from** [**http://www.jacmp.org**](http://www.jacmp.org/)
9. **Rangel A, Dunscombe P. Tolerances on MLC leaf position accuracy for IMRT delivery with a dynamic MLC [Electronic Version]. Med Phys. 2009;36(7):3304-09.**
10. **Moiseenko V, LaPointe V, James K, Yin L, Liu M, Pawlicki T. Biological consequences of MLC calibration errors in IMRT delivery and QA. Med Phys. 2012;39(4):1917-24.**

Figure 1


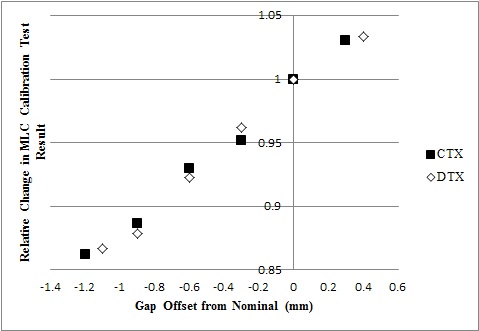


Figure 2


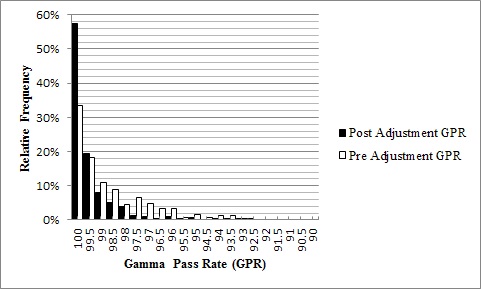


Figure 3


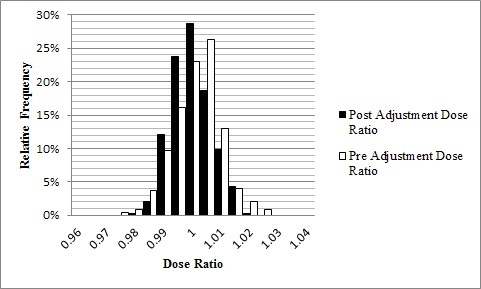


Figure Legends:

Fig. 1. Relative Change in MLC Calibration Test vs. Leaf Gap Offset. CTX and DTX and internal labels for a Varian 21Ex and 21iX LINAC respectively. Measurements were obtained by changing the leaf gap offset value with in the MLC controller. This was done to confirm that the effect of altering the leaf gap offset value has on the sweep test value is both predictable and consistent across multiple types of Varian Accelerators.

Fig. 2. Pre and Post MLC Calibration adjustment Gamma Pass Rate (GPR) Histograms. The pre adjustment GPR had a mean of 98.52 % with a standard deviation of 1.62% consisting of 247 patient specific QAs. The post adjustment GPR had a mean of 99.27% with a standard deviation of 1.17% consisting of 424 patient specific QAs.

Fig. 3. Pre and Post MLC Calibration adjustment Dose Ratio Histograms. The pre adjustment dose ratio had a mean of 0.999 with a standard deviation of 0.007 consisting of 247 unique QAs. The post adjustment dose ratio had a mean of 0.997 with a standard deviation of 0.009 consisting of 424 patient specific QAs.

| **Table 1. Pre MLC calibration adjustment IMRT QA results for three Varian accelerators.** | | | | |
| --- | --- | --- | --- | --- |
|  | Combined (σ) | LINAC 1 21Ex (σ) | LINAC 2 21Ex (σ) | LINAC 3 21iX (σ) |
| Number of IMRT QA’s | 247 | 72 | 97 | 78 |
| Mean Gamma Pass Rate % | 98.52(1.62) | 98.33(1.74) | 98.81(1.41) | 98.33(1.75) |
| Mean Dose Ratio^a^ | 0.999(0.008) | 1.004(0.008) | 0.999(0.006) | 0.992(0.008) |
| Mean Sweep Test^b^ | 0.0761(0.0011) | 0.0772(0.0007) | 0.0763(0.0008) | 0.0749(0.0002) |
| ^a^The ratio between the measured and planned doses was calculated by averaging the results from the MapCheck software’s histogram of all data points within the 10% threshold.  ^b^To measure the sweep test a Farmer-type chamber is placed at 10 cm depth in water and the reading for a 10x10 cm^2^ field given 100 MU is obtained. This reading is ratioed with reading that is taken with a dynamic MLC motion that sweeps a 0.5 cm gap over the 10 cm span. The nominal sweep test value is 0.0758. | | | | |

| **Table 2. Post MLC calibration adjustment IMRT QA results for three Varian accelerators.** | | | | |
| --- | --- | --- | --- | --- |
|  | Combined (σ) | LINAC 1 21Ex (σ) | LINAC 2 21Ex (σ) | LINAC 3 21iX (σ) |
| Number of IMRT QA’s | 424 | 79 | 176 | 169 |
| Mean Gamma Pass Rate % | 99.27(1.18) | 99.35(0.78) | 99.59(.78) | 98.92(1.51) |
| Mean Dose Ratio^a^ | 0.997(0.007) | 1.007(0.005) | 0.998(0.005) | 0.992(0.005) |
| Mean Sweep Test^b^ | 0.0756(0.0007) | 0.0751(0.0003) | 0.0756(0.0012) | 0.0762(0.0008) |
| ^a^The ratio between the measured and planned doses was calculated by averaging the results from the MapCheck software’s histogram of all data points within the 10% threshold.  ^b^To measure the sweep test a Farmer-type chamber is placed at 10 cm depth in water and the reading for a 10x10 cm^2^ field given 100 MU is obtained. This reading is ratioed with reading that is taken with a dynamic MLC motion that sweeps a 0.5 cm gap over the 10 cm span. The nominal sweep test value is 0.0758. | | | | |
